# Bone Marrow-Derived and Elicited Peritoneal Macrophages Are Not Created Equal: The Questions Asked Dictate the Cell Type Used

**DOI:** 10.3389/fimmu.2020.00269

**Published:** 2020-02-21

**Authors:** Cheryl M. Zajd, Alexis M. Ziemba, Grace M. Miralles, Terry Nguyen, Paul J. Feustel, Stanley M. Dunn, Ryan J. Gilbert, Michelle R. Lennartz

**Affiliations:** ^1^Department of Regenerative and Cancer Cell Biology, Albany Medical College, Albany, NY, United States; ^2^Department of Biomedical Engineering, Rensselaer Polytechnic Institute, Troy, NY, United States; ^3^Department of Neuroscience and Experimental Therapeutics, Albany Medical College, Albany, NY, United States

**Keywords:** peritoneal macrophages, bone marrow-derived macrophages, polarization, cytokines, phagocytosis, flow cytometry, gene expression

## Abstract

Macrophages are a heterogeneous and plastic population of cells whose phenotype changes in response to their environment. Macrophage biologists utilize peritoneal (pMAC) and bone marrow-derived macrophages (BMDM) for *in vitro* studies. Given that pMACs mature *in vivo* while BMDM are *ex vivo* differentiated from stem cells, it is likely that their responses differ under experimental conditions. Surprisingly little is known about how BMDM and pMACs responses compare under the same experimental conditionals. While morphologically similar with respect to forward and side scatter by flow cytometry, reports in the literature suggest that pMACs are more mature than their BMDM counterparts. Given the dearth of information comparing BMDM and pMACs, this work was undertaken to test the hypothesis that elicited pMACs are more responsive to defined conditions, including phagocytosis, respiratory burst, polarization, and cytokine and chemokine release. In all cases, our hypothesis was disproved. At steady state, BMDM are more phagocytic (both rate and extent) than elicited pMACs. In response to polarization, they upregulate chemokine and cytokine gene expression and release more cytokines. The results demonstrate that BMDM are generally more responsive and poised to respond to their environment, while pMAC responses are, in comparison, less pronounced. BMDM responses are a function of intrinsic differences, while pMAC responses reflect their differentiation in the context of the whole animal. This distinction may be important in knockout animals, where the pMAC phenotype may be influenced by the absence of the gene of interest.

## Introduction

Macrophages are innate immune cells that provide a first line defense against infection. While many studies historically utilized macrophage-like cell lines, the availability of knockout animals as well as development of molecular techniques for these notoriously difficult-to-transfect cells has resulted in the increased use of primary macrophages. The most commonly used primary macrophages are elicited peritoneal and bone marrow-derived. Sterile thioglycolate, injected into the peritoneum, recruits circulating monocytes that differentiate into small peritoneal macrophages. The small peritoneal macrophages are phenotypically indistinguishable from resident peritoneal macrophages (pMACs) (1). Myeloid progenitor cells, harvested from the bone marrow, are differentiated with macrophage colony-stimulating factor (M-CSF) or conditioned L929 media, to produce adherent bone marrow-derived macrophages (BMDM) (2). Neither pMACs nor BMDM preparations are homogeneous (1, 3, 4). pMACs have more lysosomal protease activity and don't significantly proliferate, indicative of a more mature phenotype (3, 5); BMDMs gravitate toward the M2 end of the polarization spectrum (6). Despite their intrinsic heterogeneity, thioglycolate-elicited pMACs and BMDMs are similar with respect to forward and side scatter as determined by flow cytometry. However, their differentiation environment may influence their phenotype, particularly if differentiation occurs in the context of a genetically manipulated (knockout or transgenic) animal. Given that pMACs and BMDMs are differentiated *in vivo* and *ex vivo*, respectively, and there *are* reported differences between the two (3, 6, 7), it is somewhat surprising that the two have not been compared with respect to the properties that define macrophages: phagocytosis, respiratory burst, polarization, and gene regulation. Despite reports that pMACs are more mature (and thus respond more robustly to stimulation), we found that BMDMs are more phagocytic (rate and amount of material ingested) and respond more robustly to polarization (surface molecule expression, gene induction/repression, and cytokine/chemokine release). These findings are consistent with the differential plasticity of pMACs and BMDMs. That is, pMACs, being differentiated *in vivo*, respond modestly when stimulated *ex vivo* while BMDMs are poised to respond rapidly and robustly to either pro-inflammatory or pro-resolving stimuli *in vitro*.

## Results

### BMDMs and pMACs Are Similar With Respect to Size and Granularity

**Figure 1:**
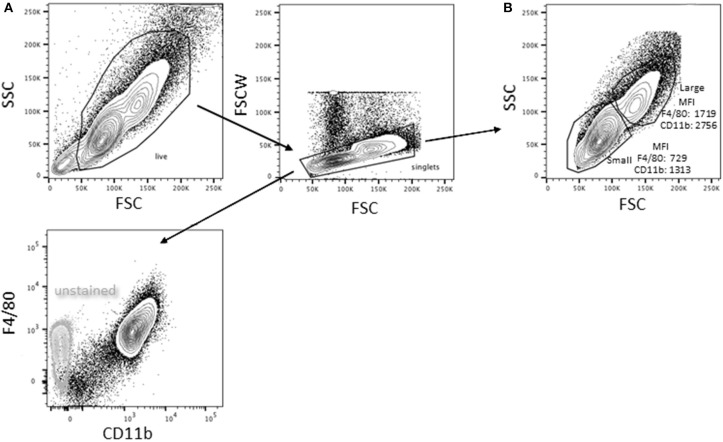
Bone marrow-derived macrophages exist as two distinct populations. Bone marrow was extracted and differentiated in L cell media as described in Methods. Adherent cells were collected 7 days post-harvesting and analyzed by flow cytometry (representative of BMDMs from 10 animals). **(A)** Virtually all (98 ± 2%) of the live singlets were CD11b^+^F4/80^+^. **(B)** After gating out dead cells/debris and selecting for singlets, two populations were identified: a minor (15.8 ± 3.4%) population of high forward and side scatter (large) cells and a major population that is smaller with lower side scatter. The large population had significantly higher expression of both F4/80 and CD11b (*p* < 0.01, *n* = 10, paired *t*-test).

Bone marrow-derived macrophages were differentiated using L-cell conditioned media as the source of macrophage colony stimulating factor (M-CSF). The resultant live, singlet population is predominantly (98 ± 2%, *n* = 8) CD11b^+^F4/80^+^ (Figure 1A); there is no detectable SiglecF or Ly6G. Based on forward and side scatter, BMDM have a minor population (15.8 ± 3.4%, *n* = 10) of large cells. As reported previously for pMACs (1), CD11b and F4/80 expression is significantly higher on large vs. small BMDM (*p* < 0.01, *n* = 10) (Figure 1B).

**Figure 2:**
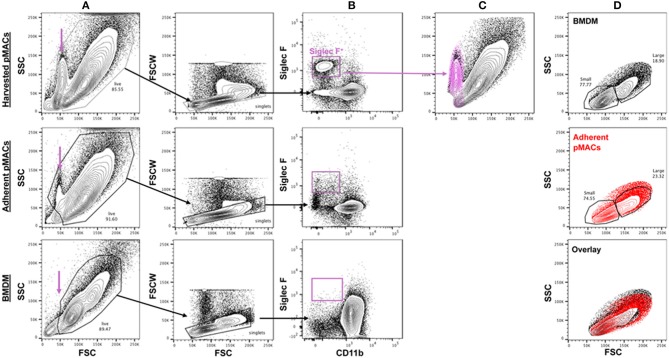
The size and granularity of bone marrow-derived and peritoneal macrophages are similar, but not identical. **(A)** Harvested peritoneal cells contain a population of small, moderately granular cells (purple arrow) that are reduced upon adhesion and not found in preparations of BMDMs. **(B,C)** Harvested peritoneal cells have a minor (11 ± 4.4%) population of Siglec F^+^ cells that is substantively removed upon adhesion (2.1 ± 1.1% post-adhesion) that co-localizes with the small, granular population. **(D)** BMDMs and adherent pMACs are similar with respect to size (FSC) and granularity (SSC). Representative of 10 preparations each of bone marrow and peritoneal macrophages.

Elicited peritoneal cells are predominantly macrophages although significant percentages of non-macrophage cells have been reported (8). Three days after injection of 3% sterile thioglycolate, peritoneal cells were harvested by lavage and the red cells lysed. One aliquot of cells was kept on ice while the other was plated in petri dishes; plates were washed after 4 h and the adherent cells recovered. An average of 5.0 × 10^7^ cells were collected (range 2.0–6.2 × 10^7^). Following adhesion, an average of 1.7 × 10^7^ cells were recovered (range 0.6–2.9 × 10^7^), an average recovery of 37 ± 10% (*n* = 15). Flow cytometry revealed a low forward scatter, moderate side scatter population in the harvested pMACs (11 ± 4.4%, *n* = 10) that was significantly diminished upon adhesion (2.1 ± 1.1%, *n* = 10, *p* < 0.01, paired *t*-test) and not present in macrophages differentiated from bone marrow (purple arrow, Figure 2A). This population was CD11b^−^SiglecF^+^, consistent with a minor contamination with eosinophils (8), a population that was substantively removed by selective adhesion (Figure 2B). Under our elicitation conditions, the (Ly6G^+^) neutrophil contamination is minimal, with an average of 1.2 ± 2% of the harvested cells *before adhesion* being CD11b^+^Ly6G^+^Ly6C^lo/neg^ (*n* = 10). The majority of recovered peritoneal cells (82.7 ± 6.2 %, *n* = 10) are CD11b^+^; this percentage rose significantly (91.5 ± 2.5 %, *p* < 0.005, *n* = 10) following adhesion (Figures 2B,C). Like BMDMs, selected pMACs contain large (~20%) and small macrophages (Figures 2A,D) (1); adhesion does not affect the relative percentages of these populations. When compared, adherent pMACs and BMDMs are similar with respect to size and granularity (Figure 2D, overlay).

The CD11b^+^ peritoneal population is Ly6C^lo^, lacks Ly6G, and is relatively homogeneous with respect to F4/80 expression (see below). Thus, selective adhesion removes the majority of eosinophils and leaves a relatively homogeneous cell population that is >90% CD11b^+^F4/80^+^Ly6C^−/lo^. Note that, from this point forward, all experiments were done with post-adherent peritoneal macrophages. For simplicity, pMACs data is presented in red and BMDM in black.

### BMDMs, but Not pMACs, Are M1 Skewed

**Figure 3:**
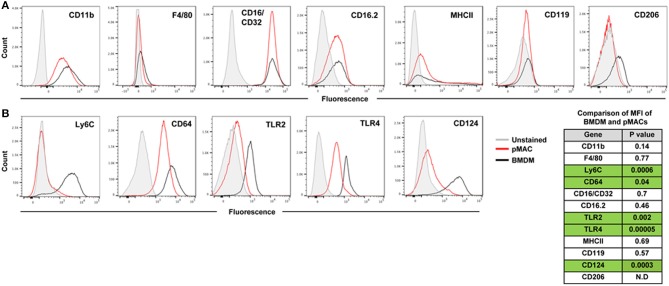
Expression of surface molecules by steady state BMDMs and pMACs. BMDMs and adherent pMACs were stained for the indicated molecules and their expression quantified by flow cytometry. Results of one BMDM and one pMAC preparation, stained on the same day, are presented. Data is presented as histograms with compensated fluorescence of the indicated antigen on the x-axis. Representative of BMDM and pMACs from 7 mice. **(A)** Antigens whose expression was not significantly different between BMDMs (black) and pMACs (red). **(B)** Antigens significantly upregulated in BMDM compared with pMACs. Table: unpaired *t*-test (*n* = 7 BMDM and 7 pMACs preparations) *p*-values for differences in antigen expression; green shading highlights significant differences, with expression in BMDMs significantly higher than pMACs. N.D. Not determined.

M1 and M2 macrophages, produced *in vitro* by IFN ± LPS and IL13/IL4, respectively, are acknowledged to be the extremes of the pro-inflammatory-to-pro-resolving spectrum (9). Physiologically, macrophages likely assume a hybrid phenotype of cell markers and cytokine/chemokine release, with their *in vivo* impact dependent on the balance between M1 and M2 outputs. At baseline, BMDMs and post-adherent pMACs have similar expression levels of CD11b, F4/80, CD16/CD32, CD16.2, MHCII, and CD119 (Figure 3A and *p* value list).

#### M1 Markers

Compared to pMACs, BMDMs express significantly higher levels of Ly6C and CD64, molecules elevated on inflammatory macrophages (10–12). The TLR2 and TLR4 pattern recognition marker receptors are more highly expressed on BMDMs (Figure 3B and *p* value list). Elevated TLR2 and TLR4 may prime macrophages for a rapid response to pathogens. While BMDMs have high Ly6C and elevated TLR2 and 4, suggestive of an M1 phenotype, their levels of MHCII expression is low and similar to pMACs. As elevated MHCII is a marker of M1 activation, its modest expression at steady state is consistent with M1 *skewing* but not bone fide activation. Thus, compared to *in vivo* differentiated pMACs, BMDM lie further toward the M1 end of the M1–M2 polarization spectrum.

#### M2 Markers

Of the three M2 markers tested, only CD124 is substantively expressed. CD119 is low, and similar, for both cell types; CD206 was variable (neg to low) (Figure 3A). As CD124 is the α chain of the IL-4 receptor, its expression could make macrophages more responsive to environmental (or *in vitro*) IL-4, an M2 polarizing cytokine.

In summary, BMDMs and (post-adherent) pMACs are similar with respect to size and granularity, expression of macrophage markers CD11b and F4/80, and three of the four Fcγ receptors (CD16/32, CD16.2) (Figure 3A). Compared to pMACs, BMDMs are Ly6C^hi^ and have elevated M1 markers TLR2, TLR4, and CD64 as well as significantly higher CD124 (Figure 3B). The expression of both M1 and M2 markers on BMDMs may prime them to polarize in response to either inflammatory or pro-resolving mediators. In contrast, pMACs express modest levels of CD64 and low levels of TLR2 and CD124, suggesting they may be more refractive to polarization. Functional assays were performed to compare BMDMs and pMACs with respect to phagocytosis, respiratory burst, and their response to polarizing conditions.

### BMDMs Are Significantly More Phagocytic Than Their pMAC Counterparts

#### *E. coli* and *E. coli*-IgG (Figure 4A)

**Figure 4:**
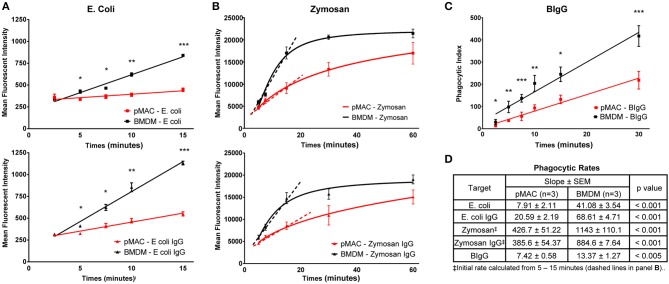
BMDMs are more phagocytic than pMACs. BMDMs and pMACs were subjected to synchronized phagocytosis using pHrodo *E. coli* ± IgG (50:1 MOI) **(A)**, Zymosan 488 ± IgG (5:1 MOI) **(B)**, or BIgG (20:1 target to cell ratio) **(C)**. **(A)** Phagocytosis was stopped at the indicated times by dislodging bound targets, diluting the sample in cold buffer, and analyzing by flow cytometry. **(B)** The fluorescence of extracellular zymosan was quenched with trypan blue immediately before analysis (*n* = 3 animals, 1 × 10^5^ cells collected/sample). **(C)** For BIgG, cells were fixed and incomplete phagosomes were detected by the addition of Alexa 488-conjugated goat anti-rabbit IgG (Invitrogen) to label the IgG on the exposed targets. The number of fully internalized targets was quantified microscopically and the phagocytic index (PI) calculated. PI = (# internal beads/# cells counted) × 100. (*n* = 3 animals, 30–40 cells per animal). **(A–C)** *p < 0.05; **p < 0.01; ***p < 0.001, unpaired *t*-test. **(D)** Composite data from 3 each pMAC and BMDM preparations reporting the rate of phagocytosis (slope of the line) and determining statistical significance using an unpaired *t*-test. Because internalization of zymosan plateaus by 30 min, an *initial* rate of phagocytosis was calculated using the 5–15 min timepoints (dashed line). BMDM are more phagocytic for all targets, regardless of the receptor used or the method used to quantify phagocytosis.

While both BMDMs and pMACs are used for phagocytosis studies, their relative phagocytic capacities have not been rigorously compared. Using pHrodo®-labeled *E. coli*, alone or IgG-opsonized (*E. coli*-IgG), we compared the phagocytic capacity of BMDMs and pMACs. pHrodo® particles are non-fluorescent when extracellular but become brightly fluorescent in the acidic environment of the phagosome. The rate of *E. coli* phagocytosis (MFI/min, slope of the time curves, Figure 4A) by BMDM was 5-fold > pMACs (41/8) and 3-fold greater for *E. coli*-IgG (69/21) (*n* = 3 BMDMs and 3 pMACs) (Figure 4D). Using an unpaired *t*-test, we determined that the rate of phagocytosis was significantly higher for BMDMs compared to pMACs (*p* < 0.001, Figure 4D).

#### Zymosan and Zymosan-IgG (Figure 4B)

A similar strategy was used to quantify internalization of zymosan and IgG-opsonized zymosan (Figure 4B). Alexa 488-conjugated zymosan ± IgG were incubated with BMDMs or pMACs. At varying timepoints (5–60 min), cell associated zymosan was detached by vortexing, trypan blue was added to quench the fluorescence of external particles, and fluorescence quantified by flow cytometry. Zymosan is considerably larger than *E. coli* and is taken up through TLR2. As zymosan phagocytosis began to plateau between 15 and 30 min, we calculated the *initial rate* of uptake using the 5–15 min datapoints. Like *E. coli*, internalization of zymosan by BMDM was significantly higher than for pMACs: 2.7-fold for zymosan and 2.3-fold for Zymosan-IgG (*p* < 0.001, Figure 4D). Notably, IgG opsonization increased the rate of, *E. coli*, but not and zymosan, internalization (Figure 4B). Noting that the rate of zymosan phagocytosis is much higher than *E. coli*, it may be that the zymosan system may be “max'd out” such that addition of IgG cannot increase the rate.

#### IgG Beads (Figure 4C)

To determine whether uptake mediated by FcγR, but independent of TLR, is different between BMDMs and pMACs, we coated 2 μm beads with (rabbit) IgG (BIgG) and calculated the rate of target internalization using synchronized phagocytosis, calculating the phagocytic index microscopically (13). The phagocytic index (number of beads/number of cells × 100) at every timepoint was significantly different, with BMDM internalizing more targets and having a phagocytic rate (slope of the line) ~2-fold higher than pMACs (Figures 4C,D).

**Figure 5:**
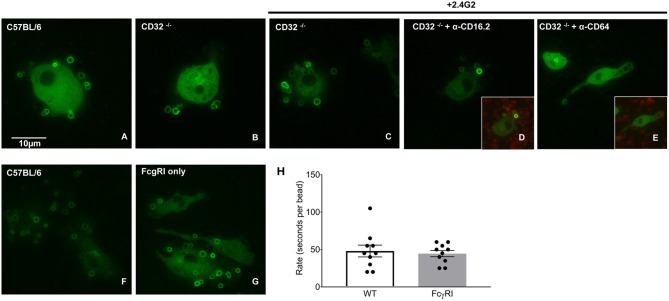
FcγRI is necessary and sufficient for IgG-mediated phagocytosis. BMDM from wild type (CD57BL/6), FcγRIIb knockout mice (CD32^−/−^) or mice expressing only FcγRI (FcγRI only) were transduced with virus encoding PKC-ε-GFP (to visualize internalization) and phagocytosis followed by live imaging as detailed in Methods. Compared to CD57BL/6 **(A)**; phagocytosis was unaffected by removal of CD32 **(B)**. Adding α-CD16/32 **(C)** or α-CD16.2 **(D)** to CD32^−/−^ cells did not affect internalization. Blocking FcγRI with α-CD64 reduced internalization **(E)** but not target binding (inset), supporting a role for FcγRI in IgG-mediated phagocytosis. **(F–H)** Internalization by C57BL/6 and FcγRI only macrophages is similar. Quantitation of phagocytic rate from movies reveal that uptake by FcγRI only cells is equivalent to controls, arguing that FcγRI is necessary and sufficient for IgG-mediated phagocytosis. **(H)** Each dot represents data from one cell, statistical significance was tested using an unpaired *t*-test.

As CD64 is the only FcγR differentially expressed on BMDMs and pMACs (Figure 3), we hypothesized that BIgG uptake requires CD64. To visualize internalization, BMDMs were transduced with PKC-ε-GFP, a molecule that concentrates at the phagosome during IgG-mediated phagocytosis (14). By using macrophages from FcγRIIb knockout (CD32^−/−^) mice, we removed the contribution of this receptor, a modification that did not substantively affect BIgG internalization (Figure 5B). Likewise, adding 2.4G2 (CD16/32 blocking antibody) to CD32^−/−^ cells did not affect phagocytosis (Figure 5C) nor did the inclusion of the 16.2 blocking antibody 9E9 (15) (Figure 5D). In contrast, the addition of α-CD64 dramatically reduced internalization (Figure 5E) with no apparent effect on binding (Figure 5E, inset). These data suggest that CD64 is the major receptor for IgG-mediated phagocytosis. To determine if CD64 is necessary and sufficient for phagocytosis, we determined the rate of BIgG internalization in BMDMs from C57BL/6 and FcγR knockout mice expressing only CD64 (FcγRI only, Figures 5G,H) (16). The fact that BMDMs from FcγRI only mice take up BIgG at the same rate as their wildtype counterparts (Figure 5H) identifies FcγRI as the major receptor mediating IgG-dependent phagocytosis. The lower expression of CD64 on pMACs provides a potential explanation for the rate differences between BMDMs and pMACs.

In summary, using three targets (*E. coli*, zymosan, and BIgG), multiple approaches (pHrodo®, Alexa 488-zymosan, and BIgG) and two readouts (fluorescence and live imaging), we have demonstrated that BMDMs are more phagocytic than pMACs. The higher rates of BMDM phagocytosis parallels the surface expression of target receptors (TLR2, TLR4, CD64, Figure 3) and is consistent with the conclusion that BMDMs are M1 skewed.

### BMDMs and pMACs Mount a Similar Respiratory Burst

**Figure 6:**
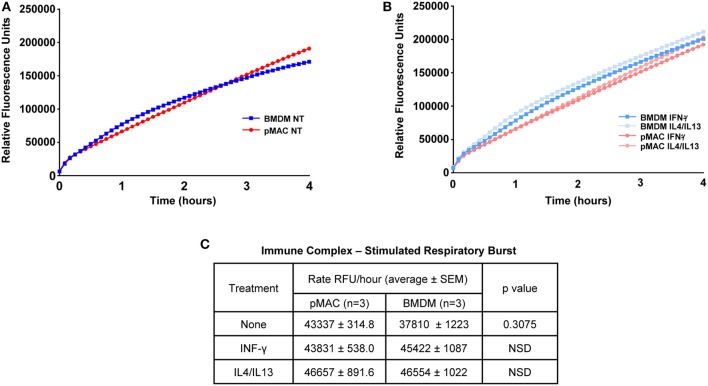
The respiratory burst is equivalent in BMDMs and pMACs. BMDMs and pMACs, untreated **(A)** or polarized overnight with IFN-γ or IL4/IL13 **(B)**, were stimulated with an empirically determined amount of immune complexes (IC) in the presence of Amplex Red®, a H_2_O_2_ reporter. Fluorescence intensities were acquired every 5 min for 4 h and the relative rate of the burst determined from the slope of the line **(C)**. Data is presented as mean ± SEM for pMACs and BMDM from 3 animals at the lowest dose of three doses of IC tested; two higher doses increased fluorescence but did not produce any difference in the burst. NSD = not significantly different.

As M1 polarized macrophages mount a larger respiratory burst than non-polarized or M2-polarized cells (17), and BMDMs are M1 skewed, we predicted that BMDMs would have a larger respiratory burst than pMACs. To test this, macrophages were incubated with immune complexes (IC) in the presence of Amplex Red®, a membrane impermeant indicator that fluoresces when oxidized. Fluorescence measurements were taken every 5 min for 4 h. Surprisingly, there was no difference in between the curves over the first 60 min with a slight divergence at later times (Figure 6A). This was not a function of “maxing out” the system as three concentrations of IC were tested (the lowest shown) and, while there was a dose dependent increase in fluorescence with increasing IC, the rate of the burst (the slope of the line) in BMDMs and pMACs were not different (Figures 6A,C). As M1 polarization increases the respiratory burst in BMDM (Supplemental Figure 1), we asked if polarization would reveal a difference between pMACs and BMDM. Cells were polarized with IFN-γ (M1) or IL4/IL13 (M2) and the respiratory burst followed with time. As with unpolarized macrophages, there were no differences in the rate of the burst under either polarization condition (Figures 6B,C).

### BMDMs and pMACs Respond Differently to Polarization

The plasticity of BMDMs is well-documented (18, 19) but how pMACs respond to polarizing cytokines is less well-studied. Thus, pMACs and BMDMs were treated with M1 (IFN-γ) and M2 (IL4/IL13) polarizing cytokines and surface molecule expression, mRNA levels, and secreted cytokines were quantified; untreated BMDMs and post-adherent pMACs served as the control (M0) macrophages. To ensure reproducibility, experiments were repeated 2–3 times, with each trial containing both BMDMs and pMACs.

#### Surface Molecule Expression (Figure 7)

**Figure 7:**
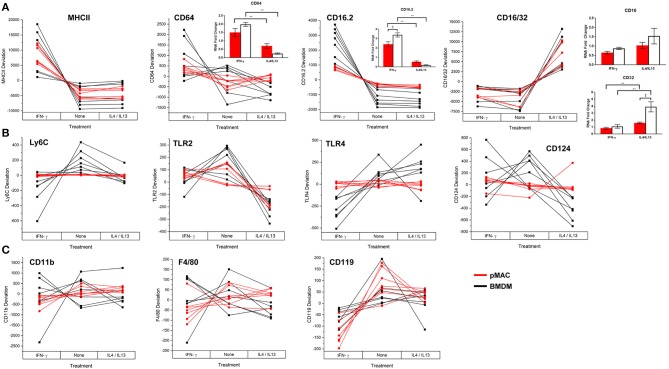
BMDM and pMACs respond differently to polarizing cytokines: surface molecule expression. BMDM and pMACs were treated with IFN-γ or IL4/IL13 for 24 h, stained for the indicated antigens, and analyzed by flow cytometry. Each line represents a single animal's cells under the three conditions. Results are reported as the within animal deviation of the measurement from the means of that animal's cells under the three conditions. This essentially removes the animal-to-animal variance and considers the within animal response. Interactions are apparent when the pattern of the responses differs between the BMDM (black) and pMAC (red) lines. Genes are loosely grouped: **(A)** genes validating polarization, **(B)** polarization dependent gene expression, and **(C)** genes whose expression is independent of cell type and polarization state. Insets: Message levels for the α chains of the Fc receptors were quantified by qPCR following cytokine treatment. The data were normalized to β-actin and the fold increase over untreated cells was calculated using the ΔΔCt method. Statistical significance was determined by ANOVA. Data for α chain PCR are presented as mean ± SEM (*n* = 3 BMDM and 3 pMACs). Daggers indicate significance based on cell type: ^‡^*p* < 0.05. Asterisks indicate differences based on polarization conditions: *p < 0.05, **p < 0.005. In general, BMDM responses are more robust than those of pMACs. pMACs and BMDM from *n* = 7 animals were analyzed.

Flow cytometry was used to quantify expression of surface molecules. After gating out debris and aggregates, fluorescence presented as Gaussian curves (Figure 3) allowing the comparison of mean fluorescent intensities (MFI) for the populations. To visualize how BMDM and pMACs respond to polarization, and to remove differences due to animal-to-animal variability, we analyzed the cells from each animal independently. That is, for each set of cells, we averaged the MFIs for each protein over the three conditions (no polarization, IFN-γ, and IL4/IL13) and plotted the difference from that mean (Figure 7); no change from the average would be plotted as “0” (i.e., MFI for each condition is the same). Thus, each line in Figure 7 is essentially a repeated measures ANOVA, the responses of the cells from a single animal over the three conditions. M1 polarization by IFN-γ is validated by the elevated expression of M1 marker genes MHCII, CD64, and CD16.2 (Figure 7A and Table 2 Polarization Main Effect) (15, 20). Similarly, IL4/IL13-dependent upregulation of CD16/32 (Figure 7A) and *downregulation* of TLR2 (Figure 7B and Table 2 Polarization Main Effect) and Arg-1 mRNA (see below) confirms M2 polarization (21). Other surface markers tested include CD11b, F4/80, and Ly6C as well as TLR4 and receptors for polarization cytokines IFN-γ (CD119) and IL4 (CD124) (Figures 7B,C). IFN-γ- and IL4/IL13- treated macrophages and their non-treated controls were analyzed for differences due to cell type (BMDMs vs. pMACs, irrespective of treatment, Cell Type Main Effect in Tables 2–4), treatment (IFN-γ, IL4/IL13, and control, irrespective of cell type, Polarization Main Effect Tables 2–4), and both (that is, do BMDMs and pMACs respond differently to polarization?, Interaction Effect Tables 2–4). By these criteria, the expression of Ly6C, TLR2, TLR4, CD16.2, and CD124 is significantly different between BMDMs and pMACs, evident from the separation of BMDM (black) and pMAC (red) lines (Figure 7) and the statistical significance (bold entries) of the cell type main effect (Table 2). CD64 expression approaches, but does not reach, statistical significance (*p* = 0.077, Figure 7A, Table 2).

**Table 1:** List of Antigens, genes, and released proteins used in these studies.

		**Flow antibodies**			**qPCR primers**		**Multiplex**
**Molecule**	**Alternate name**	**Antibody**	**Clone**	**Company**	**Sense**	**Antisense**	
CD16	FcγRIII	CD16/32-PECy7	2.4G2	BD Pharmingen	ACTGTCCAAGACCCAGCAACTAC	GCACATCACTAGGGAGAAAGCA	
CD32	FcγRIIb				GCCAAAACTGAGGCTGAGAATAC	CAGGGCTTCGGGATGCT	
CD64	FcγRI	CD64-PE	290322	R&D Systems	AGATGCTGGATTCTACTGGTGTGA	TGTGAAACCAGACAGGAGCTGAT	
CD16.2	FcγRIV	CD16.2-BV421	9E9	Biolegend	ACAAATCTTCAGCATCCTTTCGTAT	CGGTGGAAACATGGATGGA	
CD119	IFN-γ Receptor	CD119-PE	2.00E+02	eBiosciences	—	—	
CD206	Mannose Receptor	CD206-FITC	MR5D3	AbD Serotec	—	—	
CD124	IL-4R (α subunit)	CD124-PECy7	I015F8	Biolegend	—	—	
MHCII	MHC Class II	MHCII (I-A/I-E)-APC	M5/114.15.2	eBiosciences	—	—	
Ly6C		Ly6C-PECy7	HK1.4	Biolegend	—	—	
Ly6G		Ly6G-APC	1A8	Biolegend	—	—	
SiglecF	CD170	SiglecF-PE	E50-2440	BD Pharmingen	—	—	
F4/80	Adgre1	F4/80-FITC	A3-1	AbD Serotec	—	—	
CD11b	β_2_ Integrin (α chain)	CD11b-eFluor 450	M1/70	eBiosciences	—	—	
TLR2	CD282	TLR2-FITC	6C2	eBiosciences	—	—	
TLR4	CD284	TLR4-APC	SA15-21	Biolegend	—	—	
CCL2	MCP-1	—	—	—	—	—	+
CCL5	RANTES	—	—	—	—	—	+
CCL11	Eotaxin				—	—	+
CXCL1	KC	—	—	—	—	—	+
Arginase I	Arg-1	—	—	—	GGAAAGCCAATGAAGAGCTG	GCTTCCAACTGCCAGACTGT	
Inducible nitric oxide synthase	iNOS	—	—	—	TCTATCAGGAAGAAATGCAGG	CACCAGCTTCTTCAATGTGG	
Interluekin-1β	IL-1β	—	—	—	AATGAAAGACGGCACACCC	GCTTGTGCTCTGCTTGTGA	+
Interleukin-6	IL6	—	—	—	AAGACAAAGCCAGAGTCC	CCTTCTGTGACTCCAGCTT	+
Interleukin-10	IL10	—	—	—	TGTGAAAATAAGAGCAAGGCAGTG	GCCTTGTAGACACCTTGGT	+
Interleukin-12 p40	IL12 (p40)	—	—	—	AGCACTCCCCATTCCTACTT	CACGCAGACATTCCCGCC	
Interleukin-12 p70	IL-12 (p70)	—	—	—	—	—	+
Tumor Necrosis Factor α	TNF-α	—	—	—	—	—	+
β-actin		—	—	—	TTCCAGCCTTCCTTCTTGG	AGTAATCTCCTTCTGCATCC	

**Table 2:** Comparison of surface expression in BMDM and pMACs in response to polarization.

**Protein**	**Cell type main effect**	**Polarization main effect**	**Interaction effect**
CD11b	0.213	0.343	0.698
F4/80	0.910	0.438	0.189
Ly6G	0.393	0.229	0.914
Ly6C	**0.009**	**0.005**	**0.008**
MHCII	0.813	**<0.001**	0.867
TLR2	**0.002**	**<0.001**	**0.040**
TLR4	**<0.001**	**<0.001**	**<0.001**
CD64	0.077	**0.001**	0.112
CD16/32	0.802	**<0.001**	0.819
CD16.2	**<0.001**	**<0.001**	**<0.001**
CD119	0.836	**<0.001**	0.080
CD124	**0.001**	**0.008**	**0.005**

*BMDM and pMACs were polarized as described in Methods. Cell surface expression was quantified as Mean Fluorescent Intensity by flow cytometry as in Figure 3 and the statistical significance based on cell type (irrespective of polarization), polarization (irrespective of cell type), or cell type and polarization (interaction effect) determined. Specifically, the deviation from the mean, induced by IFN-γ, IL4/IL13, or left untreated (NT) was calculated for each animal (and graphed in Figure 7). Statistical significance was determined by 2-way ANOVA, thus removing the animal-to-animal variability and considering only the within-animal responses. p-values are presented. Statistically significant differences are indicated in bold text. n = BMDM and pMACs from 7 animals*.

**Table 3:** Comparison of mRNA expression from BMDM and pMACs in response to polarization.

**Gene**	**Cell type main effect**	**Polarization main effect**	**Interaction effect**
IL-12 p40	0.382	**0.035**	0.358
IL-10	0.099	**0.019**	**0.001**
Arg-1	0.757	**<0.001**	**<0.001**
iNOS	0.868	0.088	0.752
IL-6	0.185	0.395	0.644
IL-1β	**0.017**	0.088	0.178
TGF-β	0.444	0.405	0.990
CD206	0.283	**<0.001**	0.233

*BMDM and pMACs were polarized as described in Methods and expression of the indicated genes quantified by qPCR and reported as fold change (Figure 7). After logarithmic transformation, those data were fit with a linear model and statistical significance determined by ANOVA (α = 0.05) based on cell type alone (irrespective of polarization), polarization only (irrespective of cell type), or a differential response dependent on both cell type and polarization conditions (interaction effect). p-values are presented. Statistically significant differences are indicated in bold text. n = BMDM and pMACs from 4–6 animals*.

**Table 4:** Comparison of cytokine/chemokine release in BMDM and pMACs in response to polarization.

**Protein**	**Absolute concentration**			**Normalized protein release**		
	**Cell type main effect**	**Polarization main effect**	**Interaction effect**	**Cell type main effect**	**Polarization main effect**	**Interaction effect**
IL-10	**<0.001**	**0.001**	**0.001**	**<0.001**	**<0.001**	**<0.001**
IL-12 p40	**0.02**	**0.001**	0.664	0.574	**<0.001**	0.545
IL-6 (NSD from 1)	0.362	0.247	0.692	0.281	0.092	0.959
CXCL1/KC	0.386	**<0.001**	**0.026**	**<0.001**	**<0.002**	**<0.003**
CCL5/RANTES	**0.019**	0.082	0.635	0.077	**0.016**	0.528
CCL2/MCP-1	**0.002**	**<0.001**	**<0.001**	0.105	**<0.001**	**<0.001**

*BMDM and pMACs were polarized as described in Methods, the media was collected and secretion of the indicated genes quantified by Multiplex®. The released protein (Figure 9) was either fit with a linear model (Left columns) or normalized to their respective controls, logarithmically-transformed, and fit with a linear model (Right columns) and analyzed by ANOVA (α = 0.05). Significance was based on cell type (irrespective of polarization), polarization (irrespective of cell type), or a differential response dependent on both cell type and polarization (interaction effect). Statistically significant differences based on cell type, polarization, or both are indicated in bold text. n = BMDM and pMACs from 4–7 animals. The interaction significance indicates that BMDM and pMACs respond differently to polarization. Of the genes tested, the interaction effect for IL-10, CXCL1, and CCL2 is significant (bold text)*.

FcγRIIb (CD32) and FcγRIII (CD16) are detected by a single antibody, 2.4G2 (designated CD16/32 in Figure 7A). Thus, the increase in 2.4G2 staining with M2 polarization (Figure 7A) could be due to elevated expression of one or both receptors. To identify the receptor(s) that are upregulated upon M2 polarization, BMDMs and pMACs from 6 animals (3 BMDMs, 3 pMACs) were polarized with IFN-γ or IL4/IL13 and the α chains of CD64, 32, 16, and 16.2 were quantified by qPCR; control cells were left untreated. Consistent with the flow data, mRNA for CD64 increased in response to IFN-γ with no difference between BMDMs and pMACs (CD64 Figure 7A, inset). Similarly, CD16.2 was significantly higher in M1 compared to M2 cells (CD16.2 Figure 7A, inset). CD32, *but not CD16*, message was higher in IL4/IL13-treated cells with BMDMs having a significantly higher expression compared to pMACs (CD16/32, Figure 7A, inset). As CD16 message was not altered by polarization, we conclude that the increase in 2.42G signal in IL4/IL13 treated cells is due to upregulation of CD32. This is not surprising as FcγRIIb is an inhibitory receptor and M2 polarization reduces inflammation and promotes resolution.

The regulation of five genes (Ly6C, TLR2, TLR4, CD16.2, and CD124) is a function of both cell type and polarization conditions (Interaction Effect, Table 2). While these genes are known to be regulated by polarization (Table 2, Polarization Main Effect), this data demonstrates that pMACs and BMDM *respond differently* to polarization. Comparing the BMDM (black) with pMAC (red) lines (Figure 7), we find that the expression of many genes, (e.g., Ly6C, CD64, TLR 2, TLR4, CD16.2, CD124) are relatively unaffected by polarization for pMACs (i.e., red lines are relatively horizontal compared to black lines), leading us to conclude that pMACs are less responsive to their environment than BMDMs. Of the 11 genes tested, CD11b, F4/80, and CD119 are unaffected by polarization or cell type (Figure 7C, Table 2); Ly6G expression was low/neg.

#### Relative Gene Expression (Figure 8)

M1 and M2 polarized macrophages release pro-inflammatory and pro-resolving cytokines, respectively, to sustain or dampen immune responses. Given the responsiveness of BMDMs to IFNγ and IL4/IL13 (Figure 7, Table 2), we predicted that polarization would elicit a greater change in gene expression in BMDMs compared to pMACs. To test this, BMDMs and pMACs were treated as above and their mRNA subjected to qPCR for IL12/iNOS and IL10/Arg-1 (the canonical proteins/cytokines expressed by M1 and M2 cells, respectively) as well as IL6 and IL-1β (associated with inflammation) and TGF-β and CD206, selectively expressed by M2 cells.

**Figure 8:**
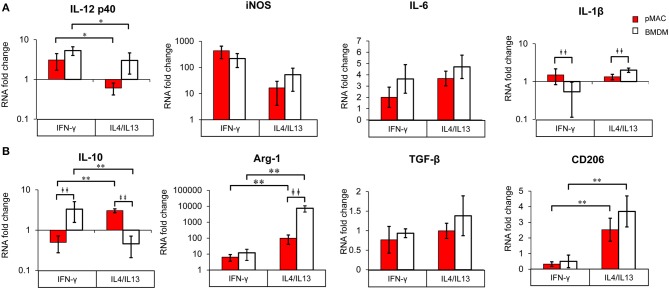
BMDM and pMACs respond differently to polarization; relative gene expression. BMDM (white bars) and pMACs (red bars) were polarized with IFN-γ or IL-4/IL-13 as in Figure 7. RNA was extracted and subjected to qRT-PCR for the indicated genes. Expression for each gene was normalized to β-actin (ΔCt) and the RNA fold change determined using the ΔΔCt method (ΔCt gene after polarization-ΔCt M0 control). Genes are loosely grouped into M1 **(A)** and M2 **(B)** markers. After logarithmic transformation, the data were analyzed and statistical significance determined by linear regression. Daggers indicate significance based on cell type (^‡^*p* < 0.05, ^‡^^‡^*p* < 0.005). Asterisks denote differences based on polarization conditions: *p < 0.05, **p < 0.005).

Due to biological variability, mRNA expression in polarized samples was normalized to their respective (untreated) controls and significance determined using linear regression. As with surface expression (Table 2), the results were analyzed to assess differences due to cell type, polarization, and interaction effect (Table 3).

#### M1 Markers (Figure 8A, Table 3)

Not surprisingly, IL12 p40 message increased significantly (3–5-fold) in response to IFN-γ, while IL4/IL13 had little effect (Polarization Main Effect Table 3); there was no significant difference between BMDM and pMAC levels of IL12 p40 mRNA (cell type effect, Table 3). While there was no difference in iNOS message between BMDMs and pMACs under either condition, iNOS expression trended lower in IL4/IL13 treated cells, approaching but not reaching, statistical significance (Table 3). This is consistent with the reported decrease in macrophage iNOS upon alternative activation (10). For IL6, there was no difference in response between the two cell types under either M1 or M2 polarizing conditions. For IL-1β, the overall change in expression was modest (<2-fold) but significantly different between BMDMs and pMACs (Cell Type Main Effect, Table 3) with the fold change in pMACs lower than BMDMs (Table 3). The low IL-1β message, coupled with no detectable protein release (by multiplex, see below) suggests that the differences in message may not be not physiologically relevant.

#### M2 Markers (Figure 8B, Table 3)

Message levels for IL10 were significantly different between BMDMs and pMACs with pMACs, but not BMDMs, showing the predicted pattern (i.e., low with IFN-γ, high in response to IL4/IL13). Statistical analysis confirmed a Polarization Main Effect (Table 3). Not surprisingly, Arg-1 expression was relatively low (and similar) in IFN-γ treated cells; the levels increased with IL4/IL13 (Polarization Main Effect) and the expression in BMDMs was significantly higher than in pMACs upon M2 polarization (Cell Type Main Effect). Thus, the polarization effect is significant as is the Interaction Effect (that is, BMDMs and pMACs respond differently to polarization, Table 3). Consistent with its designation as an M2 marker, CD206 was significantly elevated in IL4/IL13 treated cells, responding similarly in pMACs and BMDMs. Finally, there were no significant differences in message levels for TGF-β between BMDMs and pMACs or as a function of polarization conditions, although expression trended higher in response to IL4/IL13.

**Figure 9:**
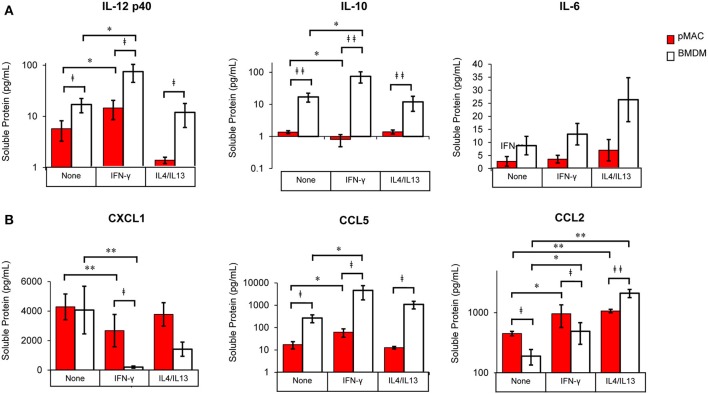
BMDM and pMACs respond differently to polarization: release of cytokines and chemokines. BMDM and pMACs were treated with IFN-γ or IL4/IL13 as in Figure 7. At 24 h, the media was collected, cells and debris removed by centrifugation, and cytokine **(A)** and chemokine **(B)** concentrations in the supernatant quantified by Multiplex®. IL-1β, IL-12p70, and TNF-α were below the limits of detection. After logarithmic transformation, data were analyzed using a linear regression model and ANOVA. Data are presented as mean ± SEM (*n* = 4–7 animals). Statistical significance between cell types is indicated by daggers (^‡^*p* < 0.05, ^‡‡^*p* < 0.005); differences due to polarization conditions by asterisks; *p < 0.05, **p < 0.005.

Table 3 presents a summary of the qPCR results (*n* = 4–6 each, BMDM and pMAC). Comparing relative mRNA levels for BMDMs and pMACs *independent of polarization* revealed that, of the eight genes tested, the expression of only IL-1β varied as a function of cell type (Cell Type Main Effect, bold text); IL10 approached, but did not reach, statistical significance. Expression of Arginase-1 (Arg-1), inducible nitric oxide synthase (iNOS), IL12 p40, IL6, TGF-β, and CD206 were not significantly different between cell types. When assessing the effects of polarization *independent of cell type*, Arg-1 and CD206 were higher upon M2 polarization while IL10 and IL12 p40 were significantly lower (Polarization Main Effect, bold text, Table 3). While not surprising for the pro-inflammatory IL12, decreased IL10 in BMDMs is inconsistent with its role as an M2 cytokine. However, this pattern tracks with the protein (Figure 9, see below) and, while the explanation isn't clear, it should be noted that other M2 markers (e.g., Arg-1 and CD206) are elevated, validating polarization. One possible explanation is that IL10 upregulation may require an additional stimulus or more time for full expression. Finally, the expression of Arg-1 and IL10 was significantly different when both cell type *and* polarization are considered (Interaction Effect, bold text, Table 3); that is, BMDMs and pMACs respond differently to polarization. Given that these experiments tested the response of BMDMs and pMACs under identical conditions, the data support the conclusion that BMDMs are generally more responsive than pMACs to polarizing environments. This is based on the fact that, for the most part, message levels in BMDMs are generally greater than their pMAC counterparts (the white bars in Figure 8 are often higher than the red bars).

As mRNA levels provide a snapshot in time, with the results being a function of message half-life and the time post-treatment, we collected the media after polarization and quantified the release of a cadre of cytokines and chemokines.

#### Cytokine/Chemokine Release (Figure 9, Table 4)

The cocktail of cytokines/chemokines released by macrophages creates the environment to which other cells (and the macrophages themselves) respond. To compare the release of chemokines/cytokines by polarized BMDMs and pMACs, cells were stimulated with IFN-γ or IL4/IL13 overnight, the media was collected, and protein release was quantified by Multiplex®. Nine chemokines/cytokines were analyzed, three of which (IL-1β, IL12 p70, and TNF-α) were below the level of detection. Under all conditions, release of IL-12p40 and IL10 was significantly higher in BMDM compared with pMACs (Figure 9A, Table 4, left, Cell Type Main Effect). Not surprisingly, IFN-γ significantly increased IL12 p40 secretion and IL4/IL13 had little effect, with the concentration of released IL12 p40 in unstimulated and IL4/IL13-treated cells being similar (Figure 9A). Interestingly, IFN-γ decreased IL10 release in pMACs but *increased* it in BMDMs (Figure 9A). IL10 secretion from IL4/IL13 exposed cells was similar to their respective no treatment controls. IL6 was not significantly different between the cell types nor as a function of polarization, although levels trended higher in BMDMs (Figure 9A). CXCL1/KC, a neutrophil chemoattractant, was similar in resting BMDMs and pMACs but was significantly lower in IFN-γ treated cells, decreasing more in BMDMs than pMACs (Figure 9B, Table 4, left, Polarization Main Effect, bold text). Release of CXCL1 upon IL4/IL13 treatment was less than untreated cells but did not reach statistical significance (Figure 9B). While the reason for the lower CXCL1 concentration under pro-inflammatory conditions (when recruitment of neutrophils would be advantageous) is not apparent, it is possible that IFN-γ upregulates CXCR1, leading to depletion of its ligand (CXCL1) from the media. Indeed, CXCR1 is upregulated on macrophages exposed to *Staph aureus* (22). CCL5/RANTES, an eosinophil, basophil, and T cell chemokine, is higher for BMDMs vs. pMACs under all conditions (Figure 9B, Table 4, left, Cell Type Main Effect, bold text). CCL5 concentrations are significantly higher in response to IFN-γ compared to no treatment, with BMDM levels higher than corresponding pMACs. CCL5 release in response to IL4/IL13 is similar to control, perhaps not surprising as it is released under inflammatory conditions. Secretion of CCL2/MCP-1, a monocyte chemoattractant, can be induced under both M1 and M2 polarizing conditions (Figure 9B). Interestingly, pMAC levels are higher than BMDM levels for untreated and IFN-γ exposed cells but BMDMs produce significantly more CCL2 than pMACs in response to IL4/IL13 (note that release is on a log scale).

**Figure 10:**
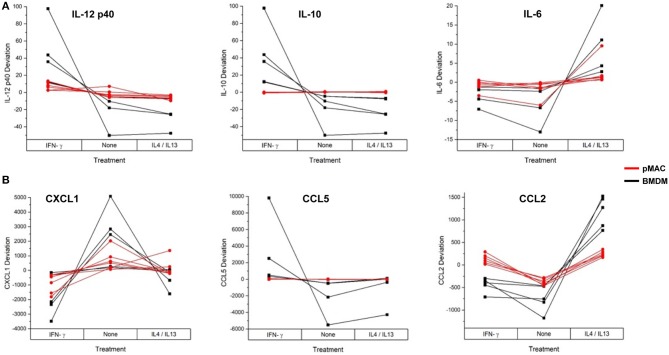
BMDM and pMACs respond differently to polarization: variations in protein release with polarization. BMDM and pMACs were treated with IFN-γ or IL4/IL13 for 24 h, the media collected, and protein release quantified by Multiplex®. The data are plotted as the deviation from the average for each treatment. Each line represents a single animal's cells under the three conditions. Results are reported as the within animal deviation of the measurement from the means of that animal's cells under the three conditions. This is essentially a repeated measures ANOVA, removing the animal-to-animal variance and considering the within animal response. Interactions are apparent when the pattern of the responses differs between the BMDM (black) and pMAC (red) curves (e.g., IL10, CXCL1, and CCL2). **(A)** cytokines and **(B)** chemokines. In general, BMDM responses are more robust than those of pMACs. *n* = 4–7 for each cell type.

These data suggest that, as with phagocytosis, surface molecular expression, and RNA levels (but not respiratory burst), BMDMs are more responsive than pMACs. To visualize the responses of the individual BMDM and pMAC preparations, we determined the mean for each animal under the three conditions (no treatment, IFN-γ, IL4/IL13) and, for each condition, calculated the deviation of that measurement from the mean (analysis similar to that in Figure 7). Comparing the responses of BMDMs and pMACs (Figure 10, black vs. red lines) it is clear that, for the cytokines/chemokines tested, pMACs are overall less responsive than their BMDM counterparts. Table 4, left, summarizes the statistical analyses with regards cytokine/chemokine release. There is a significant difference in the release of IL10, IL12 p40, CCL5, and CCL2 by pMACs and BMDM (Table 4, left, Cell Type Main Effect, bold text), with BMDM having a more robust response (red lines are relatively horizontal while black lines show dramatic variations, Figure 10). If polarization is considered irrespective of cell type, IL10, IL12p40, CXCL1, and CCL2 are differentially released (Table 4, left, Polarization Main Effect, bold text). Of interest physiologically is the interaction effect. For IL10, CXCL1, and CCL2, BMDM and pMACs respond differently, again with greater variations in release by BMDM (Table 4, left, Interaction Effect, bold text). These findings would suggest that BMDM can alter their environment to a greater extent than pMACs.

**Figure 11:**
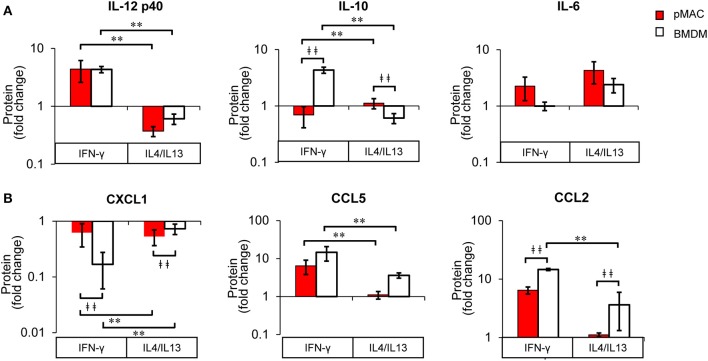
BMDM and pMACs respond differently to polarization: normalized protein secretion. Protein data from Figure 9, normalized to untreated control and reported as fold change. **(A)** cytokines and **(B)** chemokines. Data are presented as mean ± SEM (*n* = 4–7 animals per condition). After logarithmic transformation, data were analyzed using a linear regression model and ANOVA. Statistical significance between cell types is indicated by daggers (^‡‡^*p* < 0.005); differences due to polarization indicated by asterisks; **p < 0.005.

The fact that cytokine/chemokine release is basally higher in BMDMs for five of the six released cytokine/chemokines (Figure 9) makes a comparison of responses difficult. Thus, to determine the *relative* change in release as a function of polarization, we normalized the Multiplex® results for each animal to its respective non-polarized control (e.g., untreated for the same animal). From these data, we calculated a “fold change” to determine if the changes in protein release were a function of differential response to polarization or a similar magnitude of response with different baselines. Even when compensating for the lower basal levels of release by pMACs, the normalized data revealed that BMDM responses are significantly greater for IL10, CXCL1, and CCL2, trend higher for CCL5, and are not significantly different for IL12 p40 and IL6 (Figure 11). The interaction statistics for the normalized data (Table 4, right, Interaction Effect) reveal that only IL10 and CXCL1 are significantly different with BMDMs having the greater response (i.e., fold change, bold text). With respect to polarization effect, the concentrations of IL10, IL12 p40, CXCL1, CCL5, and CCL2 released are significantly different regardless of cell type. Both cell type and polarization conditions affect secretion of IL10, CXCL1, and CCL2 (Table 4, right, Interaction Effect, bold text).

Taken together, these results suggest that BMDMs (differentiated *in vitro*) are more responsive to polarizing cytokines than pMACs, making them the preferred cell type for studying macrophage plasticity. Conversely, thioglycolate elicited pMACs are differentiated *in vivo* with their phenotype being a function of their intrinsic properties *as well as* those accrued during circulation and diapedesis. The fact that expression of CD64 and TLR4 (two major signaling receptors) does not change substantively in pMACs in response to either IFN-γ and IL4/IL-13 (Figure 7) suggests that pMACs may survey their environment, having a relatively high threshold for stimulation. Quantitation of cytokine/chemokine release produced a similar response pattern (Figure 9). That is BMDM were more responsive than pMACs to polarization (red lines in Figure 10 show less deviation than the black lines representing BMDM).

In summary: Compared to pMACs, BMDM (1) are more phagocytic (Figure 4), (2) significantly upregulate surface markers in upon polarization (Table 2, Cell Type Main Effect, red vs. black lines, Figure 7) and (3) release more cytokines and chemokines (Figure 9, Interaction Effect, Table 4). The relative responsiveness of BMDM compared to pMACs suggest that they are poised to respond to infection. In contrast, pMACs have a higher threshold for response, and may serve as a “second line of defense,” acting when the threat is elevated and/or sustained.

## Discussion

Despite decades of study, much of how macrophages orchestrate innate immune responses along the pro-inflammatory to pro-resolving axis remains to be elucidated. The seminal studies on phagocytosis and respiratory burst in macrophages were done with elicited peritoneal macrophages (23–28). With the advent of cloning, macrophage biologists moved to cell lines to circumvent the difficulties in transfecting primary cells. More recently, with advances in transfection/transduction techniques as well as the realization that the changing *in vivo* environment can alter macrophage phenotype (that may not be recapitulated in cell lines), the pendulum has swung back to primary cells. Thioglycolate-elicited and bone marrow-derived are the most commonly used primary macrophages. Elicited peritoneal macrophages can be harvested 3 days after thioglycolate injection, providing a rapid and economical source of differentiated primary cells. The main disadvantage is the relatively low number of cells recovered and the fact that the macrophages are not a pure population, with the most common contaminants being neutrophils and eosinophils (Figure 2). In contrast, bone marrow-derived macrophages, differentiated from progenitor cells, are a relatively pure population that can be produced in high numbers but must be differentiated ~7 days prior to use. While both cells types are used, the rationale for using one vs. the other is often not stated. The main difference between BMDMs and pMACs is that the pMACs are differentiated in the context of the background of the mouse. Knowing that macrophage phenotype is a function of environment, we asked if there were differences between pMACs and BMDMs under controlled conditions. Searching the literature, we found few studies that compared these two commonly used cell types. To define the similarities and differences between differentiation *in vivo* vs. *ex vivo*, we directly compared elicited pMACs to BMDMs with respect to the most common metrics of macrophage function: phagocytosis, respiratory burst, and gene regulation.

Despite their similar size and granularity (Figure 2), BMDMs have higher basal expression of CD64 (the high affinity IgG receptor), TLR2, and TLR4 (Figure 3). This is notable as CD64 mediates the uptake of IgG-opsonized particles (Figure 5), and TLR2 and TLR4 are receptors for zymosan (29) and *E. coli*, respectively. Additionally, BMDMs are Ly6C^hi^, characteristic of inflammatory macrophages that are more phagocytic than their pro-resolving counterparts (30, 31). Higher expression of CD64, TLR2, and TLR4 would poise BMDMs to respond early in infections when pathogen numbers are low. Indeed, BMDMs are more phagocytic than pMACs when presented with *E. coli*, zymosan, their IgG-opsonized counterparts, or IgG-opsonized beads (Figure 4). While the higher phagocytosis and receptor expression by BMDMs is consistent with an M1 phenotype, MHCII expression is similar in BMDMs and pMACs, suggesting that BMDMs are M1 *skewed* but not M1 *activated*. The M1 skewing may result from our use of conditioned L cell media as the source of M-CSF for BMDM differentiation. While M-CSF is essential for progenitor differentiation, L cell media (LCM) contains other (undefined) factors that produce a phenotype slightly different from that produced in the presence of purified M-CSF. In our experience, BMDM differentiated in LCM produce a more homogenous cell population with more reproducible results. This agrees with anecdotal comments from online forums and macrophage colleagues that suggest that BMDMs “look better” and “proliferate better” when differentiated in LCM. Also, as BMDM were historically differentiated with LCM, using this media will allow comparisons between published data and new results. To our knowledge, a direct comparison of the phenotype of BMDMs differentiated in LCM vs. M-CSF has not been reported.

Many of the studies on IgG-dependent phagocytosis and intracellular signaling have used targets opsonized with rabbit IgG (including the targets used here, Figure 4). To our knowledge, the Fc receptor(s) utilized have not been identified [although some groups have reported that FcRIV is the major activating FcR in mice (32)]. Given that CD64 is the only FcR whose expression correlates with the increased IgG-mediated phagocytosis in BMDM (Figures 3, 4), we asked if it was the major receptor for rabbit IgG. Blocking CD64 dramatically reduced phagocytosis but not target binding (Figure 5). More definitive was the use of live imaging to quantify of the rate of phagocytosis in macrophages expressing all the Fcγ receptors (wild type) and those expressing only FcγRI. The fact that the rate of phagocytosis was equivalent argues that FcγRI is necessary and sufficient for IgG-mediated phagocytosis (Figure 5). Whether other metrics of macrophages (i.e., polarization, gene expression, respiratory burst) are CD64 dependent remains to be determined.

Given the M1 skewing of the BMDMs compared to pMACs, we expected the respiratory burst to be greater in BMDMs. We utilized Amplex Red®, a readout of H_2_O_2_ release that was stable over hours. Although immune complexes stimulated the respiratory burst, there was no difference between pMACs and BMDMs (Figure 6A). Thinking that polarization may reveal differences between the two cell types, we polarized with IFN-γ or IL4/IL13 and followed the burst with time; polarization did not produce a difference in burst between pMACs and BMDM (Figure 6B). The targets in these experiments were insoluble BSA-anti-BSA immune complexes that are small and difficult to count. Thus, we tested 1X, 2X, and 3X amounts of complex to ensure that there was a high enough “multiplicity of infection” to see a difference between the cell types. The 1X data is presented in Figure 6; the higher concentrations produced more fluorescence but no difference in the rates between BMDM and pMACs. Thus, we conclude that the higher phagocytic rates and M1 skewing of BMDMs does not correlate with increased respiratory burst, even upon IFN-γ activation (Figure 6B). Notably, pilot experiments *did* show a significant difference between pMACs and BMDMs, with pMACs having a significantly greater burst than BMDMs. However, the earlier experiments used pMACs that had not undergone selective adhesion. Selective adhesion eliminated the difference, indicating that the higher burst is likely a function of contaminating neutrophils. It is not clear why the burst would be similar if phagocytosis is greater. However, we reported that the burst is independent of phagocytosis (33), so it is likely that the extent of FcR ligation is not the rate determining step for respiratory burst.

While the first response of macrophages to pathogens is internalization and the generation of a respiratory burst, the upregulation of gene expression propagates the response, providing cytokines to which they (and bystander cells) respond. With time, the adaptive immune response is engaged, exposing macrophages to polarizing cytokines, including IFN-γ, IL4, and IL13. The question we asked was “Do BMDMs and pMACs respond similarly to polarizing conditions?” BMDMs and pMACs were tested for their response to M1 and M2 polarizing conditions (IFN-γ and IL4/IL13, respectively). Surface molecule expression (Figure 7), message levels (Figure 8), and cytokine/chemokine release (Figures 9–11) were quantified. LPS and IFN-γ are the common M1 polarization agents. We tested the effects of LPS, IFN-γ, and both on BMDM to determine our M1 polarizing conditions. BMDMs were treated overnight with LPS (100 ng/ml), IFN-γ (100 ng/ml) or both. The media was collected after 24 h and the concentration of released IL-10 and IL-12 determined by ELISA. While both LPS and IFN-γ significantly increased cytokine secretion, the combination was neither additive nor synergistic (Supplemental Figure 1A). To assess the effect of IFN-γ ± LPS on the respiratory burst, polarized cells were incubated with zymosan or zymosan IgG and Amplex Red® (as in Figure 6) and the rate of the burst calculated. Although IFN-γ significantly increased the burst LPS had no additional effect (Supplemental Figure 1B). Thus, IFN-γ alone was used for M1 polarization.

Due to the tremendous amount of data produced as well as the biological variability, the statistical analyses were complicated: differences due to cell type, polarization conditions, and the two combined had to be determined. Figures 7–11 provide a visualization of the relative response of BMDMs (black) and pMACs (red) under each condition. Based on the observation that BMDM (black lines) have greater deviations from the mean than pMACs (red lines) (Figures 7, 10) we conclude that, overall, BMDM are more responsive to their environment. This extends to phagocytosis (Figure 4). Tables 2, 4 provide the summary of statistical significance for surface molecule expression and protein secretion, respectively. Statistical significance in the “Cell Type Main Effect” column (bold text) identify molecules whose expression is different between pMACs and BMDM regardless of polarization conditions. Statistical significance in the Interaction Effect (bold text) identifies molecules whose expression is a function of both polarization *and* cell type. The conclusion from the polarization data (bold text, Tables 1–4) is that, for every measure of polarization, there are molecules whose expression is a function of cell type. Where there is statistical significance, BMDMs respond more robustly than pMACs.

Which brings us back to the fundamental question: Are BMDMs and pMACs interchangeable? We would argue the answer is no, and that the cell type chosen depends on the questions to be asked. If cell biological questions are asked, BMDMs would provide a greater range of response under most conditions (phagocytosis, gene regulation, secretion) and lend themselves to molecular manipulation. pMACs provide a readout of the responsiveness of the innate immune system in the background of the animal from which they are isolated, particularly informative for knockout or genetically modified animals. These findings raise a cautionary note that, while *in vitro* studies are informative, they do not necessarily reflect *in vivo* phenotype.

## Materials and Methods

### Buffers

Dulbecco's Modified Eagle Medium (DMEM, Gibco), ACK lysis buffer (150 mM NH_4_Cl, 10 mM KHCO_3_, 0.1 mM Na_2_EDTA, pH 7.2–7.4), Bone marrow macrophage differentiation media: DMEM supplemented with 20% L-cell conditioned media, 10% FBS, 0.2% sodium bicarbonate, and gentamycin (50 μg/ml); Macrophage media: DMEM containing 10% fetal bovine serum and gentamycin (50 μg/ml). HBSS^++^: Hanks' balanced salt solution containing 4 mM sodium bicarbonate, 10 mM HEPES, and 1.5 mM each CaCl_2_ and MgCl_2_.

### Reagents

Highly purified BSA (Cat # A0281) was purchased from Sigma. Interferon-γ (Cat # 315–05; Lot # 061398), IL4 (Cat # 214–14; Lot # 111249), and IL13 (Cat # 210–13; Lot # 111207) were purchased from Peprotech. anti-BSA IgG (Cat #B1520) was purchased from Sigma. Alexa 488-conjugated α-rabbit IgG (Cat # A11070) was from Invitrogen Life Technologies.

### Flow Antibodies (See Table 1)

Targets. pHrodo™ Green *E. coli* BioParticles™ (Cat # P35366), Zymosan A (*S. cerevisiae*) BioParticles™, Alexa Fluor™ 488 conjugated (Cat # Z23373), *E. coli* BioParticles™ Opsonizing Reagent (Cat # E2870), Zymosan A BioParticles™ Opsonizing Reagent (Cat # Z2850), and Amplex™ Red Hydrogen Peroxide/Peroxidase Assay Kit (Cat # A22188) were purchased from Life Technologies. *E. coli* and Zymosan were IgG-opsonized per manufacturer's instructions.

#### Immune Complexes (IC)

IgG immune complexes were formed by incubating 1 mol of highly purified BSA with 3 mol (rabbit) anti-BSA IgG (60 min, 37°C, with rotation). Complexes were washed with PBS before use.

#### IgG-Coated Beads (BIgG)

Were prepared as described previously (14). Briefly, 2 μm borosilicate microspheres (Duke Standards, Thermo Scientific USA) were coated sequentially with poly L-lysine, activated with dimethylpimelimidate · 2 HCl, washed, and incubated with highly purified BSA (overnight, 4°C with rotation). BSA beads were blocked (1 M Tris, pH 8.0), washed, and opsonized with rabbit anti-BSA IgG.

### Cells

Male and female C57BL/6 mice, 12–16 weeks of age, were the cell source. FcγRI-only mice (VG1505) (16) for collection of bone marrows, were provided by Regeneron Pharmaceuticals, Inc. Requests for FcγRI only mice should be sent to Regeneron, they cannot be fulfilled by the corresponding author. Animals were bred in the Albany Medical College Animal Resource Facility. All procedures were done in under NPHS guidelines using protocols approved by the Albany Medical College Institutional Animal Care and Use Committee. The sex of the animal providing the cells was recorded, we found no differences in the responses from male and female mice.

#### Bone Marrow-Derived Macrophages (BMDMs)

Bone marrow was extruded from the femurs and pelvises of euthanized mice and differentiated in bone marrow macrophage differentiation media according to published procedures (34, 35) Albanesi, 2012 #16787}. Cells were used 7–10 days after harvesting.

#### Elicited Peritoneal Macrophages (pMACs)

Elicited peritoneal macrophages were recruited and harvested according to published methods (36). Briefly, mice were injected i.p. with aged thioglycolate. After 4 days, peritoneal exudates were collected using sterile phosphate buffered saline (PBS). Red blood cells were lysed using ACK lysis buffer. For selective adhesion, cells were plated in untreated petri dishes; after 4 h, non-adherent cells removed by washing in PBS and the adherent population removed using 5 mM EDTA/PBS (15 min with agitation). Recovered cells were resuspended in HBSS^++^ and used within 4 h. Note: Due to the fact that BMDMs are differentiated *in vitro*, and pMACs are used the day of harvest harvest, the BMDMs and pMACs used in each experiment were never from the same animal.

### Phagocytosis

#### E. coli and Zymosan

For flow-based assays, 2 × 10^5^ post-adherent pMACs or BMDMs were added to flow tubes and the tubes placed on ice. Cold pHrodo™ Green *E. coli* ± IgG (50:1 MOI) or Alexa 488-conjugated zymosan ± IgG (5:1 MOI) were added; total volume of the assay was 35 μL. Tubes were kept on ice to allow target binding, then transferred to 37° waterbath to initiate a synchronized wave of phagocytosis. At varying times (2.5–15 min for *E. coli*, 5–60 min for the larger zymosan), the tubes were removed from the waterbath, vortexed to dislodge bound targets, and 100 μl of ice cold HBSS^++^ added to stop phagocytosis. Tubes were placed on ice and read as soon as possible. To quench the fluorescence from external zymosan, 4% trypan blue was added immediately before reading. Data was collected on 60–100 × 10^6^ cells/tube.

#### IgG-Coated Beads (BIgG)

Sychronized BIgG phagocytosis was done as previously described (36). Briefly, macrophages (5 × 10^4^) were plated onto coverslips in 24 well plates and cooled in an icebath. Targets (20:1) were added and allowed to bind on ice. Plates were transferred to 37°C waterbath and fixed at the indicated times. Incomplete phagosomes were detected by the addition of Alexa 488 α-rabbit IgG which labeled the IgG on the exposed targets. Cell number was determined by DAPI staining.

#### Real Time Imaging

Live imaging for determination of phagocytic rates was done as previously detailed (34). BMDM were virally transduced to overexpress PKC-ε-GFP, brought into focus on the stage of the spinning disk confocal microscope, BIgG were added, and images taken every 5 s for 10 min. The rate of phagocytosis was determined to be the number of frames from the first indentation of the plasma membrane through the first frame showing completely enclosed particles × 5 (seconds between frames).

### Respiratory Burst

pMACs and BMDMs (3 × 10^4^) were seeded in 96 well plates and allowed to adhere. The Amplex™ solutions were prepared as per manufacturer's instructions. Cells were stimulated with an empirically determined amount of immune complexes in the presence of 50 μM Amplex™ Red and 0.1 U/mL horseradish peroxidase in HBSS^++^. Plate was maintained at 37°C in a BioTek™ Synergy™ 2 Multi-Mode Microplate Reader. Data was collected every 5 min over the 4 h time period, the baseline (no treatment) was subtracted from each value and the net relative fluorescence units presented.

### Macrophage Polarization

Polarization was done on adherent macrophages (1 × 10^6^) with 100 ng/mL IFN-γ or IL4 + IL13 (25 ng/ml each) for 24 h. Cells thus polarized (and with a non-treated control) were used for flow cytometry, respiratory burst, quantitative polymerase chain reaction (qPCR), and cytometric bead arrays. While LPS + IFN-γ is often used together for M1 polarization (10), and synergize in cytotoxicity assays (37, 38), we found that a 24 h incubation with IFN-γ was sufficient to M1 polarize (Tables 2–4), with the addition of LPS not significantly enhancing the IFN-γ responses for either cytokine release nor phagocytic rate (Supplemental Figure 1). As cytotoxicity assays are done on the order of days, it may be that feedback and/or gene regulation that occurs in that timeframe contributes to synergy. Alternatively, the fact that C57BL/6 mice are more M1 skewed at steady state, perhaps IFN-γ is sufficient to upregulate M1 markers. Finally, as summarized by Jackson Labs (https://www.jax.org/news-and-insights/jax-blog/2016/june/there-is-no-such-thing-as-a-b6-mouse), all C57BL/6 sub-strains are not the same. Our mice, originally ordered from Jackson but bred in our facility over many years, apparently do not require inputs from both IFN-γ and TLR agonists for the readouts we are studying.

### Flow Cytometry

1 × 10^6^ cells were used for flow analysis on polarized cells. For all analyses except CD16/CD32 staining, cells were blocked with CD16/CD32 (Mouse Fc Block, Clone 2.4G2) for 15 min on ice, then incubated with antibodies to the proteins of interest (45 min, on ice). Unstained cells were used to establish flow cytometer settings. Fluorescence minus one (FMO) controls were used for compensation. Flow cytometric data were acquired on a FacsCalibur (Becton and Dickenson, Franklin Lakes, NJ) using FlowJo and the data analyzed using FlowJo Software

(Tree Star, Ashland, OR). Antibodies used are listed in Table 1.

### Quantitative Polymerase Chain Reaction (qPCR)

Quantitative polymerase chain reaction was conducted per previous lab protocols (39). The primers used are listed in Table 1. Expression for each gene was normalized to β-actin (ΔCt) and the RNA fold change determined using the ΔΔCt method (ΔCt gene after polarization—ΔCt M0 control).

### Cytokine Bead Array Polarization Assay

Following polarization, cell supernatants were collected, clarified by centrifugation, and stored at −80°C until analyzed. Secreted cytokine/chemokines were quantified using a Bio-Plex Pro™ Mouse Cytokine, Chemokine, and Growth Factor custom 9-plex assay (Control#64145335). The assay was run per manufacturer's instructions, using 50 μl of the supernatant. Concentrations were calculated from standard curves. The kit contained M1 markers: IL12 p40, IL-1β, IL6, IL12 p70, and TNF-α, the M2 marker IL10, and the three chemokines: CCL1/KC (neutrophil chemoattractant), CCL2/MCP-1 (monocyte chemoattractant protein), and CCL5/RANTES (T cell homing factor). The absolute protein release as well as the release normalized to non-polarized controls was determined.

### Statistical Analysis

The number of subjects required for each of the assays were estimated using power analysis (set at 80% and α = 0.05) using GPower 3.1 (Dusseldorf, Germany). Statistical significance was determined by a repeated measures analysis of variance (ANOVA). The repeated measures ANOVA was conducted with the within effect of polarization state nested within macrophage type and macrophage type as a between animal effect. For Figure 6, each antigen was quantified by flow cytometry, MFIs for each animal under each condition (no treatment, IFN-γ, and IL4/IL13) were averaged and the deviation from that mean was calculated thus effectively removing the animal-to-animal variability and considering only the within-animal response, analogous to a repeated measures design. Protein release (Figure 9) was treated similarly. Statistical significance was accepted at *p* < 0.05. Data are reported as *p*-values, with statistical significance accepted a *p* < 0.05. For qPCR and protein release (Figures 7–10), the data are reported as mean ± SEM. As the qPCR data were not normally distributed, a Generalized Linear Transform was used to fit the data to estimate parameters subsequently used in the ANOVA. For polarization (Tables 2–4), statistically significant difference based on cell type alone are shaded green, those based on polarization alone are shaded blue, and interaction differences (i.e., BMDM and pMACs respond differently to polarization) are shaded orange. Animal numbers ranged from 4 to 7, with n referring to the number of BMDM and pMACs tested. Statistical analysis was performed using Minitab (State College, PA).

It is recognized that the presentation of the data in Figures 7–11 is unfamiliar. This is due to the complexity of the data. The data assessing how BMDMs and pMACs respond to polarization are quite complex as there are two factors: cell type and polarization state. Each factor has a minimum of two levels (cell type: BMDM and pMAC and polarization state: none, IFN-gamma, and IL4/IL13). To properly assess the effect of each of these factors and their levels, both the main effect of each factor and the interaction between the two factors is assessed, as the response to polarization stimuli may vary depending on the cell type.

The statistics in Tables 2–4 correspond to Figures 8–11. They include the main effects of cell type and polarization state and the interaction effects which were computed using a Generalized Linear Transform (as opposed to a linear transformation, e.g., classical multiple regression) since the relationships between cell type and polarization state was hypothesized to be non-linear. Additionally, each biological replicate was considered to be cells from one animal, and due to animal-to-animal variability, the magnitude of responses sometimes differed between animals. To assess the effect of polarization stimuli while removing the animal-to-animal variance, changes in the polarization state of cells from each animal were analyzed using a repeated measures ANOVA where each line represents the response of cells from a single animal/biological replicate (Figures 6, 9).

## Data Availability Statement

All datasets generated for this study are included in the article/Supplementary Material.

## Ethics Statement

The animal study was reviewed and approved by Albany Medical Center Institutional Animal Care and Use Committee.

## Author Contributions

CZ and AZ contributed equally to this work. They performed the majority of the experiments, collected and analyzed data, contributed to the statistical analysis, generated many of the figures, figure legends, materials and methods, their results, and contributed to the discussion. GM and TN did some experiments, analyzed results, generated the figures, figure legends, and materials and methods. PF and SD, biostatisticians, worked with CZ, AZ, and ML to determine the best statistical methods and drove the statical analyses. RG contributed to intellectual development of the project, experimental design, and writing. ML initiated the study, coordinated the experiments, did experiments, analyzed data, wrote the manuscript and worked with PF and SD on the statistics.

### Conflict of Interest

The authors declare that the research was conducted in the absence of any commercial or financial relationships that could be construed as a potential conflict of interest.

## Supplementary Material

Supplemental Figure 1 LPS does not synergize with IFN-γ with respect to the respiratory burst or cytokine secretion. BMDM were treated with LPS (100 ng/ml), IFN-γ (100 ng/ml) or both for 24 h; controls received neither. The media was collected for quantitation of IL-10 and IL-12 by ELISA **(A)** and the respiratory burst quantified using the Amplex Red® fluorescence assay in response to zymosan or IgG-opsonized zymosan **(B)**. When treated with IFN-γ, BMDM and pMACs released equivalent concentrations to IL-10/IL-12 and produced the same amount of oxidized Amplex Red regardless of the presence of LPS. Thus, IFN-γ (100 ng/ml) was used for M1 polarization in these studies. Each symbol represents cells from one mouse. ^*^*p* < 0.05, ^**^*p* < 0.005 compared to no treatment. *p*-values were determined using one-way ANOVA and Tukey's test.
