# Supplementary figures and images for: Bone Marrow-Derived and Elicited Peritoneal Macrophages Are Not Created Equal: The Questions Asked Dictate the Cell Type Used
Source: Front Immunol. 2020 Feb 21;11:269. doi: 10.3389/fimmu.2020.00269 (PMC7047825; doi:10.3389/fimmu.2020.00269)

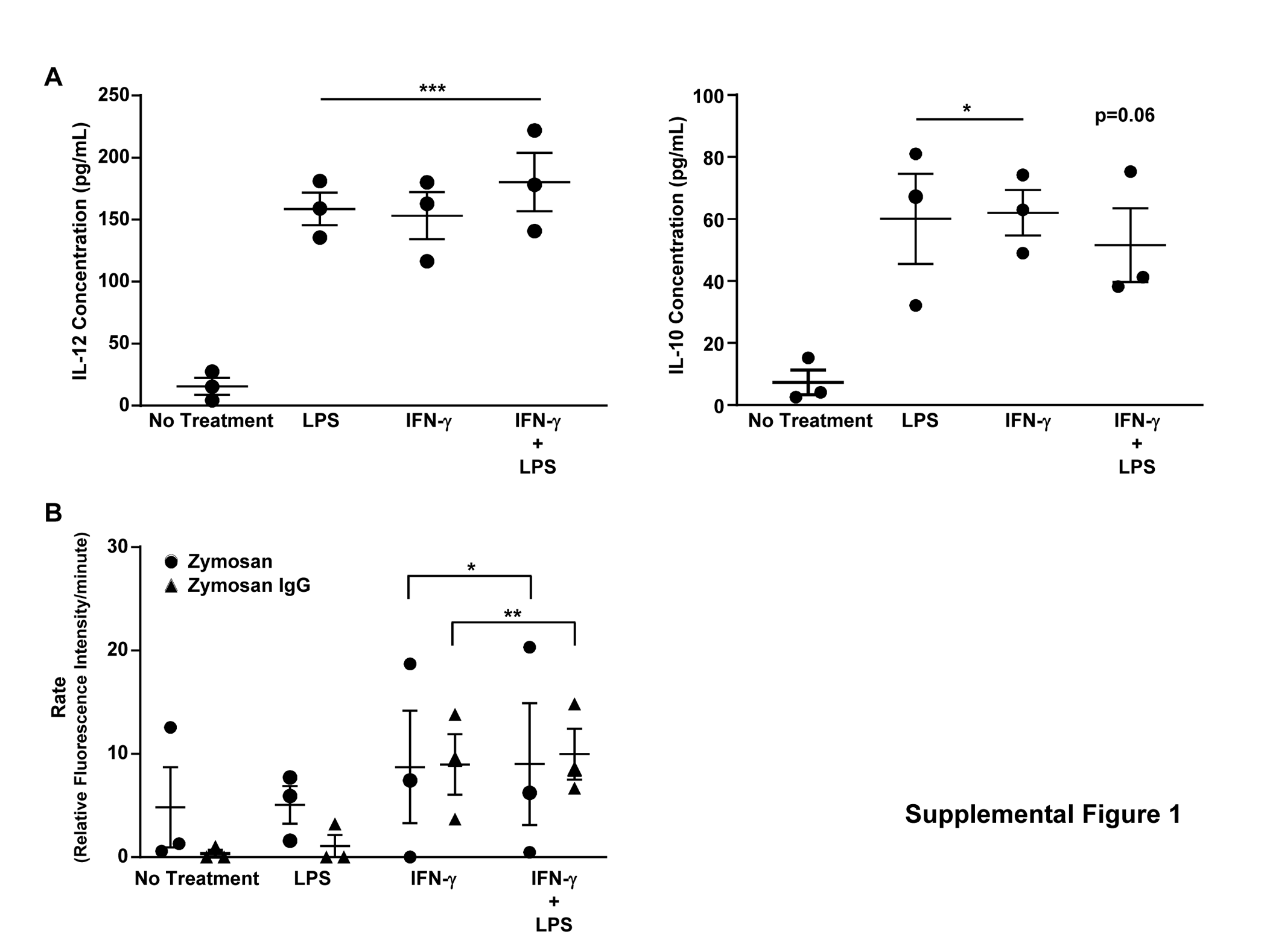

Supplement: Supplemental Figure 1 — LPS does not synergize with IFN-γ with respect to the respiratory burst or cytokine secretion. BMDM were treated with LPS (100 ng/ml), IFN-γ (100 ng/ml) or both for 24 h; controls received neither. The media was collected for quantitation of IL-10 and IL-12 by ELISA (A) and the respiratory burst quantified using the Amplex Red® fluorescence assay in response to zymosan or IgG-opsonized zymosan (B). When treated with IFN-γ, BMDM and pMACs released equivalent concentrations to IL-10/IL-12 and produced the same amount of oxidized Amplex Red regardless of the presence of LPS. Thus, IFN-γ (100 ng/ml) was used for M1 polarization in these studies. Each symbol represents cells from one mouse. *p < 0.05, **p < 0.005 compared to no treatment. p-values were determined using one-way ANOVA and Tukey's test. [file Image_1.TIF]
